# Axonal injury is detected by βAPP immunohistochemistry in rapid death from head injury following road traffic collision

**DOI:** 10.1007/s00414-022-02807-z

**Published:** 2022-04-30

**Authors:** Safa Al-Sarraj, Claire Troakes, Guy N. Rutty

**Affiliations:** 1 Department of Clinical Neuropathology, King’s College Hospital NHS Foundation Trust, London, UK; 2 London Neurodegenerative Diseases Brain Bank, Basic and Clinical Neuroscience Department, Institute of Psychiatry, Psychology and Neuroscience, King’s College London, London, UK; 3 East Midlands Forensic Pathology Unit, University of Leicester, Leicester Royal Infirmary, Robert Kilpatrick Building, Leicester, UK

**Keywords:** Forensic Neuropathology, Beta Amyloid Precursor Protein (βAPP), Axonal injury, Traumatic brain injury, Road traffic collision

## Abstract

The accumulation of βAPP caused by axonal injury is an active energy-dependent process thought to require blood circulation; therefore, it is closely related to the post-injury survival time. Currently, the earliest reported time at which axonal injury can be detected in post-mortem traumatic brain injury (TBI) tissue by βAPP (Beta Amyloid Precursor Protein) immunohistochemistry is 35 min. The aim of this study is to investigate whether βAPP staining for axonal injury can be detected in patients who died rapidly after TBI in road traffic collision (RTC), in a period of less than 30 min.

We retrospectively studied thirty-seven patients (group 1) died very rapidly at the scene; evidenced by forensic assessment of injuries short survival, four patients died after a survival period of between 31 min and 12 h (group 2) and eight patients between 2 and 31 days (group 3). The brains were comprehensively examined and sampled at the time of the autopsy, and βAPP immunohistochemistry carried out on sections from a number of brain areas.

βAPP immunoreactivity was demonstrated in 35/37 brains in group 1, albeit with a low frequency and in a variable pattern, and with more intensity and frequency in all brains of group 2 and 7/8 brains from group 3, compared with no similar βAPP immunoreactivity in the control group. The results suggest axonal injury can be detected in those who died rapidly after RTC in a period of less than 30 min, which can help in the diagnosis of severe TBI with short survival time.

## Introduction

The timing of traumatic brain injury, dependent on neuropathological examination, is one of the most challenging aspects of forensic neuropathology. It is a complex task and requires close interpretation and integration of a set of morphological and immunohistochemical tests together with other autopsy findings. For instance, the timing of intracranial haemorrhage (subdural or extradural haematoma) and contusions depends on assessing the extent of the reactive changes in the dura or brain, respectively, and the healing stages; including type of inflammatory cell infiltration (neutrophils and macrophages) in addition to changes such as the extent of necrosis and ischaemia in adjacent neural tissue.

Another level of timing of traumatic brain injury (TBI) depends on assessing the morphological representation of the axonal injury via demonstration of axonal injury by H&E stain, silver stain, and more time-accurately, by immunostaining for βAPP [1–7]. βAPP is a ubiquitous membrane glycoprotein produced in the cell body and plays a physiological role in cell adhesion and endogenous neuroprotection in response to injury (4, 8). It is transported by fast axoplasmic transport and accumulates proximal to the site of axonal injury [4, 5].The predominant mechanism in developing axonal injury in TBI is mechanical shearing and stretch forces produced by inertial acceleration/deceleration stresses to the head. This inertial loading triggers dynamic shear tensile and compressive strain within the brain [9–12]. Consequently, certain parts of the brain move at a slower pace relative to others, leading to deformation of brain tissue, putting long-tract structures such as axons and blood vessels at risk of shear injuries [13, 14]. The distortion of the axonal cytoskeleton subsequently disturbs the normal axonal transport leading to accumulation of transported axonal products in the injured region and alteration of neuronal homeostasis [15, 16]. Therefore, detection of βAPP by immunohistochemistry is indicative of disruption of axonal transport but it is not entirely specific for the cause of this disruption which could, for example, be due to trauma, ischaemia, infection or drug overdose [17–19].

BAPP accumulation is an active energy-dependent process requiring blood circulation and therefore is closely related to the patient’s post-incident survival time [20, 21]. The earliest time in which axonal injury has been reported to be detected by immunohistochemistry in autopsy brain examination is 35 min after traumatic brain injury [7, 20]. The amount of accumulated βAPP increases with the time of survival post-axonal injury, becoming easily and more frequently detected after about one hour and reaching its maximum intensity and frequency at around 24 h after head injury [3]. It is reported that there may be a correlation between the size of the accumulated βAPP immunoreactivity and period of survival, but this doesn’t enable precise timing of injury despite using morphometrical assessments of the size of the accumulated protein [6]. After 24 h, the staining of the βAPP starts to fade, appearing as faint accumulation and acquiring a more granular pattern with the passage of time. Ultimately it disappears from the site of axonal injury after 30 days leaving faint granular deposits in glial cells [2]. The activation of microglia cells at the site of axonal injury is another useful tool in timing of TBI. Microglial cells can become activated at the site of the axonal injury clearly at around 36–48 h and can be useful in timing axonal injury when closely correlated with the site of axonal injury as detected with βAPP [22, 23].

Within our forensic neuropathology practice, we observed a few cases of head injury with βAPP immune reactivity in which the survival time is reported to be less than 30 min and in others with rapid and near instantaneous death. The question has thus arisen as to what is the possible minimum post-incident time of survival that axonal injury can be detected by βAPP immunohistochemistry. This is an important question in medico-legal practice for the diagnosis of traumatic brain injury in patients who died instantaneously/rapidly after head injury and also would shed light on the pathophysiology of axonal injury in severe head trauma.

The aim of this work was to investigate if βAPP staining for axonal injury can be detected in the autopsy brain in patients with rapid death following fatal RTC. By answering this question, the study provides further insight into the rapidity which βAPP can be demonstrated after fatal head injury and may provide further understanding into the pathophysiology of axonal injury.

## Materials and Methods

### Case selection

Forty-nine consecutive cases of adolescents and adults dying following a road traffic collision (RTC) and one single occupancy fatal air crash were referred to the East Midlands Forensic Pathology Unit and authorized for invasive autopsy examination by HM Coroner. In each case, the relatives of the deceased consented for samples of the brain to be retained for the study purposes (REC code 04/Q2501/64). There were no study exclusion criteria. The autopsies were comprehensive including examination of the body, internal organs and the brain and spinal cord (when indicated). They were undertaken in line with the following standards: 1) Council of Europe Committee of Ministers—Recommendation R (99) 3 of the committee of ministers to member states on the harmonization of medico-legal autopsy rules 1999; 2) Code of practice and performance standards for forensic pathology in England, Wales and Northern Ireland. Royal College of Pathologists, UK. 2012.

All case histories were assessed by one of the authors (GR) who is senior forensic pathologist specialized in the investigation of road traffic fatalities and a pre-hospital doctor for the regional ambulance service. In this later capacity, he regularly attends life changing, life threatening, and fatal road traffic incidents where he assesses and treats the complex trauma presented to him on a time critical basis. The assessment of the cases in terms of the pathology present and the timeframe for potential survival was made using the combined experience of these two roles.

The details of the circumstances of the incident, date, location, and time of alert to the emergency services as well as the time pronounced dead were recorded in an Excel spreadsheet, along with the presence or absence of medical intervention, the cause of death and nature of the head and cervical spinal findings at autopsy. The autopsy report was reviewed to consider the extent and nature of all the injuries sustained in the incident, including the presence or absence of bleeding associated with organ trauma.

For the analysis, the subjects were then divided into three different groups depending on the time of the survival after the traumatic incidence. A fourth group of non-traumatic rapid deaths was used as a control group.

The details of the cases are summarized in Tables 1, 2.

### Group 1 (rapid death)

We want to investigate if axonal injury can be detected earlier the previously reported 35 min [20]. For purpose of this article, we define rapid death as when the period of survival is estimated to be a few to several minutes but less than 30 min which will give us wider window of time (but less than 35 min that is reported before) to accommodate the estimated shorter timing as in some cases the exact timing may not be possible to ascertain. This group comprised of those who died at the scene of the incident. Thirty-seven (37/49) victims were known or considered, based on witness accounts or the pathology identified at autopsy, to have died rapidly at the scene of the incident. These comprised of 6 females and 31 males with an age range of 14–90 years (Table 1).

Establishing the exact time of death in RTC is not an easy task and cannot be completely accurate in most cases. There is understandably a period of time between alerting and travelling of emergency services to the scene of the accident, and assessment of the victim before pronouncement of death, which in many cases could have occurred much earlier than officially documented. Therefore, we rely upon critical forensic examination to assess if pathological findings are compatible with a period of survival. The deaths were considered very rapid if the injuries sustained were incompatible with life and any period of survival in victims with marked disruption of the cranium, chest or abdominal organ or internal decapitation (base of skull/C1 or C1/C2) of the upper cervical spine. In this group, were also those victims who died rapidly, i.e., within a few minutes based on the observations of those attending the scene or on the pathology identified at autopsy, for example a completely transacted thoracic aorta, avulsion of the heart or lack of cavity blood accumulation in the presence of gross organ trauma (for example major liver laceration with only smearing of peritoneal cavity blood) (Table 1).

Head injury was the principal cause of death in 11/37 patients. In a further seven, the head injury was combined with at least one other major injury to another anatomical area, for example spine, chest, or abdomen as the cause of death. For the other 19/37 cases, an injury to a part of the body other than the head was considered the principal injury that caused death; however, features of traumatic brain injury were also present although they are not considered the main cause of death. Looking at features of head trauma, thirty-five patients (35/37) are recorded to have head injuries of varying degree with features including scalp bruising, skull fracture, subdural haematoma, and subarachnoid haemorrhage. The remaining two cases (case 20 and 21) died mainly from chest injuries with no visible traumatic brain injury. Nevertheless, head injury of some degree caused by acceleration/ deceleration is likely to be present considering the type of the RTC (one was a pedestrian and the other was the driver, both involved in high-speed collisions) 2).

**Table 1:** Details of cause of death and evidence of rapid death ( death at the scene) for group 1, and the recorded timing of survival according to the hospital notes for group 2 (short survival time) who survived between 31 min and 12 h after the RTA and group 3: (long survival time) comprised who had survived between 2 to 31 days after the RTA3.

Group 1: (rapid death at the scene)		
	**Cause of death**	**Evidence of rapid death for few to several minutes but less than 30 min**
1	Chest injuries and abdominal injuries	Laceration of the heart, transacted aorta with severe abdominal injuries but without bleeding consistent with few minutes survival
2	Head, chest and spinal injuries	Exact time of incident is unknown. There is small amounts of bleeding to injuries so a few minutes survival is expected
3	Head, chest and abdominal injuries	Very severe chest and abdominal injuries including major vessel avulsion and tears bu there are no significant haemorrhages, therefore consistent with rapid death
4	Head, chest and abdominal injuries	Severe chest injuries. The patient suffered cardiac within few minutes at scene
5	Severe chest, abdominal and head injuries	Lacerations of heart, lung, liver, spleen and major blood vessels. Reported rapid cardiac arrest at the scene
6	Chest abdominal and pelvic injuries	Full thickness lacerations of the heart, liver and spleen with small amounts of bleeding consistent with few minutes’ survival
7	Chest injuries	Severe chest injuries with torn pericardium, ruptured heart and blood in chest consistent with few minute survivals. There is lack of blood to abdomen with liver tears support this view
8	Severe head injury	The driver’s car was rotating and then rolled, when the door came out and his head struck the carriageway sustaining severe head injury, fracture of sternum, laceration of the lung but with small amounts of bleeding consistent with survival of few minutes
9	Severe Chest and abdominal injuries	Liver lacerations, spinal injuries at C1,C2, fracture of sternum and pelvis, extensive liver lacerations but small amounts of bleeding consistent with survival of few minutes
10	Severe Head injury and abdominal injuries	Severe head injury and absence of bleeding in association with abdominal organ injuries consistent with survival of few minutes
11	Severe head injury	Extensive head injury with cerebral hemispheres avulsed from brainstem which incompatible with life therefore death was rapid and expected in few minutes
12	Severe head and neck injuries	Severe neck injuries and decapitation at C1 level which are care incompatible with life and so death wasSy rapid expected to be of few minutes
13	Severe head and chest injuries	Severe thoracic and abdominal injuries with minimal blood including a near complete avulsed heart, therefore death was very rapid and expected within few minutes
14	Chest injuries	Transected aorta with chest blood as well as a liver laceration with small amounts of bleeding, therefore consistent with rapid death within few minutes
15	Head injury and multiple organ injuries	Extensive multi system organ injury including large liver laceration with small amounts of abdominal bleeding consistent with rapid death of a few minutes
16	Severe head, chest and abdominal injuries	Significant abdominal trauma including marked lacerations of liver and ruptured diaphragm with small amounts of bleeding consistent with rapid death of few minutes
17	Severe abdominal injuries and head injuries	Severe abdominal trauma including liver lacerations with 2.5 L of blood in abdomen. Death took minutes for blood to accumulate in abdomen which is consistent with rapid death of few minutes.
18	Severe head injury	The extensive abdominal and head injury incompatible with life so virtually death was rapid and expected within few minutes
19	Severe chest injuries	Transected aorta and inferior vena cava with blood in pleural cavity consistent with rapid death of only few minutes
20	Severe chest injuries	Transected aorta and lacerations of liver and spleen with blood in pleural and abdominal cavities consistent with rapid death of few minutes only
21	Severe head injury	23 min between incident and pronouncement by paramedics of death. But due to the severity of the head injury and lack of bleeding in abdomen considering liver injury death was expected to be extremely rapid and in keeping to be within few minutes
22	Severe chest injuries	Due to the extensive nature of the chest injuries including a transected aorta with little blood loss and displaced heart to right pleural cavity death would be very rapid and in keeping to be within few minutes
23	Postural asphyxia due to inverted position resulting from a road traffic collision	The victim was in pulseless Electric Activity (PEA) within 3 min of incident and declared dead
24	head injury and chest and abdominal injuries	Lack of bleeding in the chest and abdominal cavity despite major skeletal and organ trauma including lacerations of liver, spleen and kidneys death is expected to be rapid in keeping to be within few minutes
25	Chest injuries and intoxication with ethanol, mephedrone and amphetamine	Major aortic tear with associated haemopericardium in keeping with rapid death of a few minutes only
26	Head, chest and abdominal injuries	Due to the severity of the injuries and minimal bleeding especially in abdomen with severe liver lacerations and lack of significant bleeding, death was expected to be very rapid and of few minutes
27	Neck/chest/abdominal injuries	Injuries with C1/C2 injury, transection of spinal cord, laceration of liver and spleen, raptured diaphragm and 1 L of blood in pleural cavity rapid death was expected to be in a few minutes
28	Severe chest and abdominal injuries including liver lacerations	Death announced at the scene – There was very little blood associated with liver lacerations, fractured sternum and cavity injuries supports rapid death and in keeping to be within few minutes
29	Severe neck injury	Survival was recorded about 3 min. This is an asphyxia type death from steering wheel crushing the laryngeal cartilage causing upper airway obstruction
30	Severe chest injuries including partial transection of aorta	Complete aortic transection with haemothorax consistent with survival of a few minutes only
31	Severe chest injuries including lung lacerations and aortic rupture	Split pericardium, tears in left ventricle, transection of aorta, tears in liver. Due to severity of chest, cardiac and aortic injuries, and death expected to be rapid and in keeping to be within few minutes
32	Severe head and chest injuries	Severe spinal cervical injury and lack of bleeding from abdominal and chest injuries death is expected to be rapid and in keeping to be within few minutes
33	Severe head injury	Dislocation fracture of C1 and transection of the spinal cord. Due to nature and extent of injuries, death is expected to be rapid and in keeping to be with few minutes
34	Chest injuries	severe traumatic chest injury with aortic tear, severe thoracic spine injury and bilateral pleural cavity bleeds. The victim was in cardiac arrest at scene within few minutes
35	Head injury and spinal injuries	Head injury with severe thoracic spinal and rib fractures and spinal cord injury death is expected to be rapid in keeping within few minutes
36	Chest and neck injuries	Transected aorta and full thickness laceration of the left ventricle but only associated with small amounts of bleeding. Death is expected to be rapid
37	Severe Neck injury	Sever nature of neck and spinal cord injury consistent with very rapid death of few minutes
**Group 2: (short survival time) comprised who survived between 31 min and 12 h after the RTA**		
	**Cause of death**	**Evidence of survival time from**
		**hospital data**
1	Chest and pelvic injuries and head injury	11 h and 56 min
2	Head injury	10 h and 50 min
3	Abdominal aortic rupture and head injury	1 h and 31 min
4	Head and pelvic injuries	7 h and 49 min
**Group 3: (long survival time) comprised who had survived between 2 to 31 days after the RTA**		
	**Cause of death**	**Evidence of survival time from**
		**Hospital data**
1	Chest injuries and head injury	4 days
2	Hypoxic/ischaemic brain injury secondary to head injury	3 days
3	Propofol Syndrome and head injury	3 days
4	Pneumonia complicating head injury	
	31 days	
5	Head injury	11 days
6	Head injury	13 days
7	Pulmonary embolism due to deep venous thrombosis due to immobility secondary to head injury	2 days
8	Head injury	2 days

### Group 2 (short survival time)

This group comprised of four male patients aged 26–78 years who had a well-documented survival period between one hour 31 min and 12 h after the RTC. All the patients had features of traumatic brain injury. The main cause of death was recorded in these four cases as head injuries, abdominal injuries, neck injuries and chest and pelvic injuries (Tables 1 and 2).

### Group 3 (long survival time)

This group comprised of eight patients (six males and two females) aged between 61 and 87 years who had survived between 2 and 31 days after the RTC. Head injury was the main, or a contributor to the, cause of death in 6/8 cases. Two patients died of brain ischaemia due to systemic injuries (Tables 1 and 2).

### Group 4 (control group)

We selected control cases in which have certain information of sudden death comprised of seven patients (two males and five females) aged between 18–72 years. Four patients have died from sudden unexpected death in epilepsy (SUDEP). The mechanism of death in SUDEP is poorly understood but it includes mechanism such as autonomic and brainstem dysregulation with cardiac arrhythmia in which the death is expected to be quick and within few minutes.

The other three control brains are from patients or died suddenly due to underlying peripheral cancer (samples were provided by the London Neurodegenerative Diseases Brain Bank). One patient with epilepsy was reported to show nodular grey matter heterotopia and the brains of two patients (72 and 69 years) showed mild aging processes, including restricted abnormal phosphorylated Tau immunoreactivity in the hippocampus and entorhinal cortex corresponding to Braak stage II. There were no features of head injury, ischaemia or inflammation in any of the seven brains (Table 2).

**Table 2:** Details of studied cases including main autopsy, post-mortem interval and summary of brain pathology and βAPP results in the white matter (WM), corpus callosum (CC), internal capsule (IC), cerebellum (Cb) and brain stem (BS) divided in three groups; Group 1: (rapid death) comprised of those who died at the scene of the incident in few to several minutes but less than 30 min, Group 2: (short survival time) comprising those who survived between 31 min and 12 h after the RTA, Group 3: (long survival time) comprising those who survived between 2 and 31 days after the RTC and Group 4 (control) comprising patients who died from sudden unexpected death in epilepsy (SUDEP) or died rapidly and unexpectedly following cancer diagnosis

Group 1: (rapid death) comprised of those who died at the scene of the incident in few to several minutes but less than 30 min											
	**Age/ Sex**	**Head and neck autopsy findings**	**Post-mortem interval**	**CNS findings**	**Brain weight**	**βAPP**					
						**WM**	**CC**	**IC**	**Cb**	**BS**	
1	47 M	Scalp bruises	84 h	Thin SDH, SAH	1380 g	+	+	+	-ve	-ve	
2	47 M	Scalp haemorrhage, periosteal haemorrhages	37 h	SAH, thin SDH, basal brain contusions ( frontal and temporal)	1080 g	+	+	+	-ve	+	
3	21 M	Large scalp laceration, skull fracture (frontal and parietal bones)	64 h	SAH	1550 g	+	+	+	-ve	+	
4	51 M	Scalp bruises	46 h	SAH	1350 g	+	+	+	+	+	
5	56 M	Deep scalp bruises, basal skull fractures	72 h	SAH	1500 g	+	+	+	+	+ + +	
6	56 M	Scalp bruises	72 h	SAH	1410 g	+	+	+	+	+ +	
7	26 M	Skull fracture left orbital bone	82 h	Thin SDH, SAH	1628 g	+	+	+	-ve	-ve	
8	51 M	Scalp laceration, multiple fractured skull,	72 h	SAH, brain contusions	1480 g	+	+	+	+	+	
9	48 F	Scalp bruises	92 h	Thin SAH	1230 g	+	+	+	-ve	-ve	
10	55 M	Diffuse scalp haemorrhages, multiple skull fracture including hing skull fracture ( fatal single occupancy airplane crush)	48 h	SAH	1460 g	+ +	+	-ve	+	-ve	
11	49 M	Extensive scalp haemorrhages, hinge/base /plate skull fractures, C1fracture	13 h	SAH, cerebral hemispheres avulsed from brainstem	1400 g	+	+	+	+	-ve	
12	24 M	Scalp laceration,, hinge skull fracture, internal decapitation at C1	57 h	Diffuse SAH	1400 g	+	+	-ve	-ve	-ve	
13	65 M	Scalp haematoma, complex skull fracturs, lower cervical spine fracture	34 h	Patchy SAH	1200 g	+	+	+	+	+	
14	51 M	Scalp bruises	72 h	SAH, contusion	1450 g	+ +	+ + +	+ +	+ +	+ + +	
15	18 F	Scalp bruises	96 h	SAH, multiple brain contusions	1300 g	+	+	+	+	+	
16	21 M	Scalp bruises and laceration, C1 fracture with widening of space between this and skull	72 h	SAH, epidural haemorrhage, spinal cord injury	1200 g	+	+	+	-ve	-ve	
17	18 F	Deep scalp bruises	72 h	SAH, contusions	1200 g	-ve	-ve	+	-ve	+ +	
18	14 F	Scalp bruises, multiple skull fractures,	24 h	Severe brain lacerations and contusions, SAH and multiple small intra cerebral haemorrhages	1300 g	+	+	+	-ve	+	
19	25 M	None	38 h	Unremarkable	1600 g	+	+	+	-ve	-ve	
20	41 M	T11,T12 vertebral fractures	45 h	Unremarkable	1380 g	+	+	+	-ve	-ve	
21	60 M	Hinge skull fracture, spinal fracture	47 h	extensive SAH, spinal cord injury	1450 g	+	+	+	-ve	+	
22	67 M	Facial skeleton fracture	69 h	Unremarkable	1750 g	+	+	+	-ve	-ve	
23	30 M	spinal fracture at C5 level	96 h	Thin SDH over cerebrum and SDH at C5,6	1550 g	-ve	+	+	-ve	-ve	
24	21 F	Skull fracture in the occipital bone extending to base of skull	58 h	SAH, petechial brain haemorrhages, brainstem haemorrhages	1310 g	-ve	+	-ve	-ve	-ve	
25	26 M	scalp bruises	48 h	Large SDH,	1450 g	-ve	-ve	-ve	-ve	-ve	
26	25 M	Deep scalp bruises	34 h	Brain haemorrhages including petechial haemorrhages in cerebral white matter, cerebellum and braistem	1410 g	-ve	+	+	-ve	-ve	
27	60 M	Extensive scalp, facial injuries, haemorrhage around upper cervical spine. Soft tissue disruption between C1 and C2	55 h	SAH	1350 g	-ve	+	+	-ve	-ve	
28	34 M	Central forehead, bridge of nose and philtrum bruises due to frontal facial impact	43 h	SAH and thin SDH	1464 g	-ve	+	+	-ve	-ve	
29	33 M	Scalp bruises	63 h	SAH	1290 g	-ve	+	+	-ve	-ve	
30	21 M	Scalp swelling across the vertex	NA	SAH	1450 g	+	-ve	+	-ve	-ve	
31	39 M	Left parietal periosteal bleed	35 h	Epidural haemorrhage and SAH	1590 g	+ +	+	+	-ve	-ve	
32	54 M	separation of soft tissues between C1 and C2, odontoid peg fracture	120 h	Thin SAH, brain stem haemorrhages, contusions, tear of corpus callosum, contusion to temporal lobe,	1500 g	-ve	+	-ve	-ve	-ve	
33	18 F	hinge fracture, C1 and base of skull dislocation	96 h	SAH, Ponto-medullary contusion	1340 g	+	+	+	-ve	+	
34	90 M	Posterior scalp injury, thoracic spine injury	131 h	SAH	1457 g	+	+	+	-ve	-ve	
35	55 M	Scalp bruises,	29 h	SAH, brain contusions in the occipital lobe	1200 g	-ve	-ve	-ve-	-ve	+	
36	32 M	Scalp bruises,, internal decapitation at c1/c2 and fracture in upper thoracic spine	55 h	Diffuse SAH,	1700 g	-ve	-ve	-ve	-ve	-ve	
37	52 F	posterior scalp haemorrhage, depressed skull fracture right frontal region with extension,, internal decapitation with C1 dislocation as well as C7 fracture	69 h	Diffuse SAH	1240 g	+	Ve	-ve	-ve	-ve	
**Group 2: (short survival time) comprising those who survived between 31 min and 12 h after the RTA**											
	**Age/ Sex**	**Head and neck autopsy findings CNS**	**Survival**	**PM Interval**	**Summary of Brain autopsy findings**	**Brain weight**	**βAPP**				
							**WM**	**CC**	**IC**	**Cb**	**BS**
1	65 M	Scalp bruises, C4/5 fracture	14 h	120 h	Brain swelling SDH, SAH, spinal haemorrhages	1500 g	+ +	+ +	+ +	-ve	-ve
2	26 M	Scalp bruises	11 h	53 h	Brain contusion, SDHs, SAH, brain swelling	1540 g	+	+	+	+	+ + +
3	78 M	Scalp bruises	31 min	68 h	Mild SAH on the temporal lobes, cerebellum and base of brain extending to upper cervical spinal cord	1480 g	+	+	+	+	+
4	79 M	Scalp bruises,	8 h	60 h	SDH, SAH, white matter haemorrhages in the frontal and temporal lobes	1250 g	+ + +	+ +	+	+	+ + +
**Group 3: (long survival time) comprising those who survived between 2 and 31 days after the RTC**											
	**Age/ Sex**	**CNS/Head**	**Survival**	**PM interval**	**CNS autopsy findings**	**Brain weight**	**βAPP**				
							**WM**	**CC**	**IC**	**Cb**	**BS**
1	41 M	displaced spine fracture	4 days	31 h	SDH, oedema,	1500 g	+	+	-ve	-ve	+
2	55 M	Scalp bruises	3 days	111 h	Intra cerebral Haemorrhages, ischaemia/hypoxia, swollen brain	1430 g	-ve	+	+	-ve	+
3	16 F	Scalp bruises, occipital bone fracture,	3 days	47 h	SAH, SDH	1560 g	+	+ +	+	+ +	+
4	63 M	Head injurycraniotomy	31 days	144 h	brain swelling,	1450 g	-ve	+	+	-ve	+
5	87 M	Scalp bruises	10 days	120 h	Temporal lobe contusions SAH, SDH and intra cerebral haemorrhages	1360 g	+ +	+ + +	+ + +	-ve	-ve
6	81 F	Scalp bruises,	13 days	120 h	brain atrophy	1030 g	+	-ve	-ve	-ve	-ve
7	47 M	Scalp bruises,	2 days	58 h	brain contusion and haemorrhages	1400 g	+ + +	+ + +	+ +	+	+
8	65 M	Scalp bruises, skull fracture,	2 days	126	SAH, SDH and contusions	1650 g	+ + +	+ + +	+ +	+ +	+ +
**Group 4 (control) comprising patients who died from sudden unexpected death in epilepsy (SUDEP) or died rapidly and unexpectedly following cancer diagnosis**											
	**Age/sex**	**Circumstances**	**CNS**	**Survival**	**Brain wt**	**PM interval**	**βAPP**				
							**WM**	**CC**	**IC**	**Cb**	**BS**
1	18 F	Found dead; history of repeated epilepsy episodes	Unremarkable	Found dead	1275 g	61 h	-ve	-ve	-ve	-ve	-ve
2	49 M	Witnessed epileptic fit, collapsed, cardiac arrest	Unremarkable	Rapid after epileptic fit within minutes	1352 g	54 h	-ve	-ve	-ve	-ve	-ve
3	40 F	Cardiac respiratory arrest after witnessed epileptic fit	Nodular grey matter hetero	Rapid after epileptic fit within minutes	1215 g	78 h	-ve	-ve	-ve	-ve	-ve
4	24 M	Found dead in bed, known history of chronic epilepsy	Unremarkable	Found dead assuming rapid death after epileptic fit	1326 g	66 h	-ve	-ve	-ve	-ve	-ve
5	72 F	Metastatic carcinoma of unknown origin to the Lung and liver	Unremarkable	Found dead in the hospice	1328	47 h	+				
Non Specific	-ve	-ve	-ve	-ve							
6	69 F	Recently diagnosed with a pancreatic body carcinoma with liver metastases	Unremarkable	Deteriorated and died in short time	1288	58 h	-ve	-ve	-ve	-ve	-ve
7	51 M	Lung adenocarcinoma developed pleural and cardic effusion	Unremarkable	Rapidly deteriorated and died	1386	33 h	-ve	-ve	-ve	-ve	-ve

## Neuropathology examination

In all the cases, the brains were examined by one of the authors who is a senior forensic pathologist (GR) as part of the police and coronial examination. A standardized examination and sampling protocol was undertaken in each case. The brains were dissected at the time of autopsy examination by cutting into coronal sections of 1 cm thickness. The slices were closely examined according to the anatomical regions and then a full set of tissue blocks taken for histology, including two sections of cerebral cortex and white matter from central semi ovale, two levels from corpus callosum, basal ganglia including internal capsule, cerebellum (and its white matter) and brainstem including midbrain and pons (total 9 tissue blocks) [24].

### Tissue preparation

Formalin-fixed, paraffin-embedded tissue (FFPE) blocks were used for this investigation. Brain autopsy samples were fixed in 10% formalin for a minimum of 48 h. Tissue processing and immunohistochemical stains were then carried out following standard methods.

All sections produced were stained with βAPP by means of automated IHC staining, using the Leica bond platforms.

The following primary antibody for βAPP was used; Millipore MAB348 diluted to 1/5000. ( detailed methods of tissue fixation, processing and immunohistochemistry are available in appendix1).

The sections were examined by using high magnification X400 and X630 to assess the immunoreactivity of βAPP by two pathologists who were blinded to the case number, diagnosis and period of survival. The examination was conducted twice by each pathologist to record BAPP accumulated deposits in the axons of white matter, and the average number of βAPP accumulated deposits per 10 × High Power field in each block was recorded, with random selection of the sections between cases to reduce observer bias. In cases in which there is difference in the counts between the two pathologists, a repeat count is taking after discussing the case. However, there was no significant inter- or intra-examiner disagreement because the majority of cases have low frequency of βAPP-accumulated deposits.

In cases of rapid death and short survival, the βAPP deposits are tiny and infrequently scattered, particularly in cases of very rapid death where it was only possible to record their presence or absence. In cases in which the survival time was more than 30 min, the βAPP immunostaining was more clearly deposited and therefore further assessed by their shape and pattern in addition to their frequency and extent of distribution. A semi-quantitative method using a three-point scale was used to assess the βAPP immunoreactivity with amount being classified as low ( +) when they are 1/10 × high power field (HPF) or less, medium (+ +) when they are 2–5 /10 × HPF and high (+ + +) frequency when they are more than 5/10xHPF.

The selection of blocks, method of preparation, staining and assessment of the control samples was carried out in the same method as for the other test groups.

## Results

Forty-nine cases were recruited into the study; 48 had died following a road traffic collision and one following a single occupancy air crash. These are divided to three groups (the results are summarized in Table 2):

### Group 1 (rapid death)

The brains from 35 out of 37 patients in this group showed BAPP immunoreactivity in one or more brain regions. βAPP was demonstrated in one of the five brain regions in three victims and in two or more regions in the other 32 patients. The corpus callosum was the most frequently affected region (32 cases) with the cerebellum and the pons the least affected areas (9 and 14 cases, respectively).) The two brains which were negative for βAPP staining in this group (cases 24 and 36) were from a patient who died of chest injuries and alcohol intoxication and from another who died from severe neck injury/decapitation of the head at level of C1/2. The βAPP was also positive in two patients (case number 20 and 21) who had only chest injuries but with no visible traumatic brain injuries. However, a degree of head injury caused by an acceleration and deceleration mechanism is likely as both had a significant impact being involved in a high-speed collision as a pedestrian or driver.

**Fig. 1:**
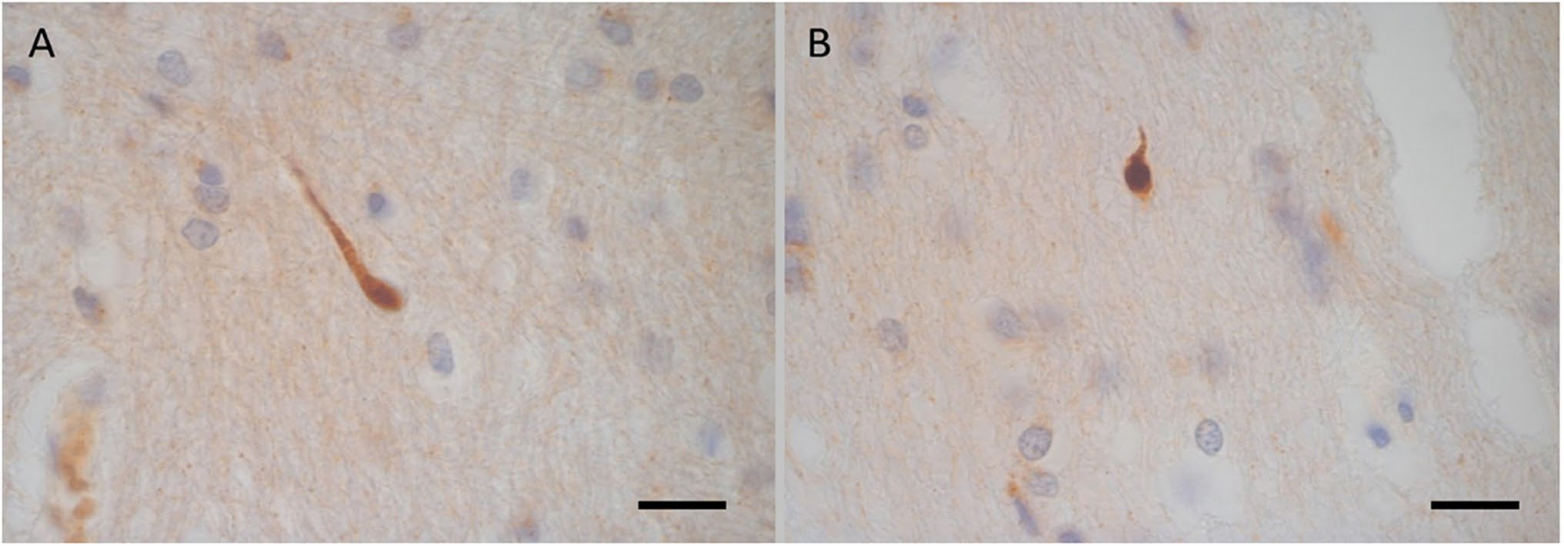
Case 15 group 1. 18-year-old female. Driver of car in an RTC. Died at the scene. The deceased had TBI with subarachnoid haemorrhages, brain contusions and multi-organ injuries. She was considered to have died instantaneously / near instantaneously. There is a small focus of βAPP immunoreactivity in the corpus callosum (X630) as a small globule representing early axonal swelling attached to a short axonal staining

**Fig. 2:**
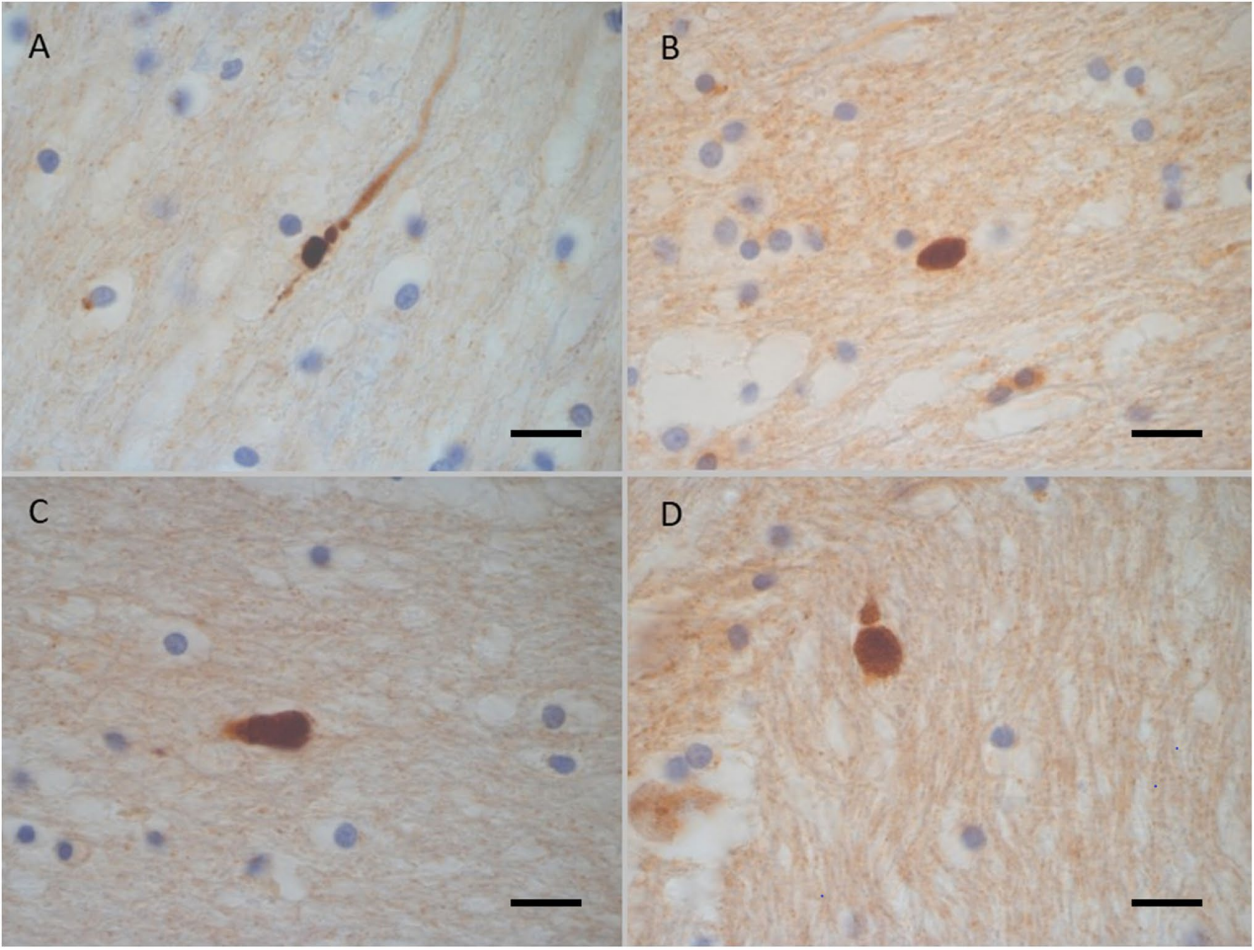
Case 16 group 1. 21-year-old male. Driver of a car in an RTC. Died at scene. The deceased had head, chest and abdominal injuries. Death was considered to have been rapid if not near instantaneous based on the injuries sustained. There are small foci of βAPP immunoreactivity (X1000) in the corpus callosum (**A**), Cerebral white matter (**B**), Cerebellar white matter (**C**) and pons(**D**)

The βAPP staining showed a variable pattern and frequency of immunoreactivity (Table 2). The immunoreactivity was seen as scattered small, well-defined and individually located tiny globules, in some cases attached to a thin filamentous structure representing the site of an early axonal injury (Fig. 1). But the majority of βAPP immunostaining was seen as minute dot-like structures which can only be detected with careful screening of the sections using high-power microscopic examination (Fig. 2). The βAPP deposits were seen infrequently ( +) and estimated as about or less than 1/10 × high power field. They were also present in regions not adjacent to haemorrhages.

### Group 2 (short survival time—30 min to 12 h)

**Fig. 3:**
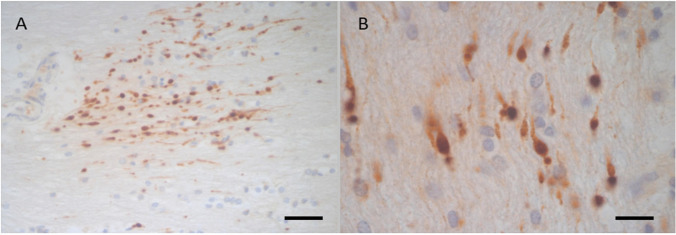
Case 4 group 2. 79-year-old pedestrian. Taken to hospital with head injury (SDH, SAH, WM haemorrhages). Died 8 h after incident. There are more frequent (Fig A X400) and more intensely stained (Fig B X 1000) βAPP accumulations in the corpus callosum compared with the case in Fig. 1 and 2

All the brains from the three victims in this group were positive with βAPP and showed more frequent accumulation in all the examined regions (including the cerebellum and pons) than those seen in group 1, with moderate and high frequency (+ + , +  + +) in at least one region (Table 2). The βAPP deposits were seen as well-defined rounded or fusiform dense globules or as a thickened filamentous pattern that were easily recognized in the microscopic examination (Fig. 3). They were more frequent and densely stained in cases with more than 1–2 h survival time compared with those of less than one hour.

### Group 3 long survival time – (2 to 31 days)

**Fig. 4:**
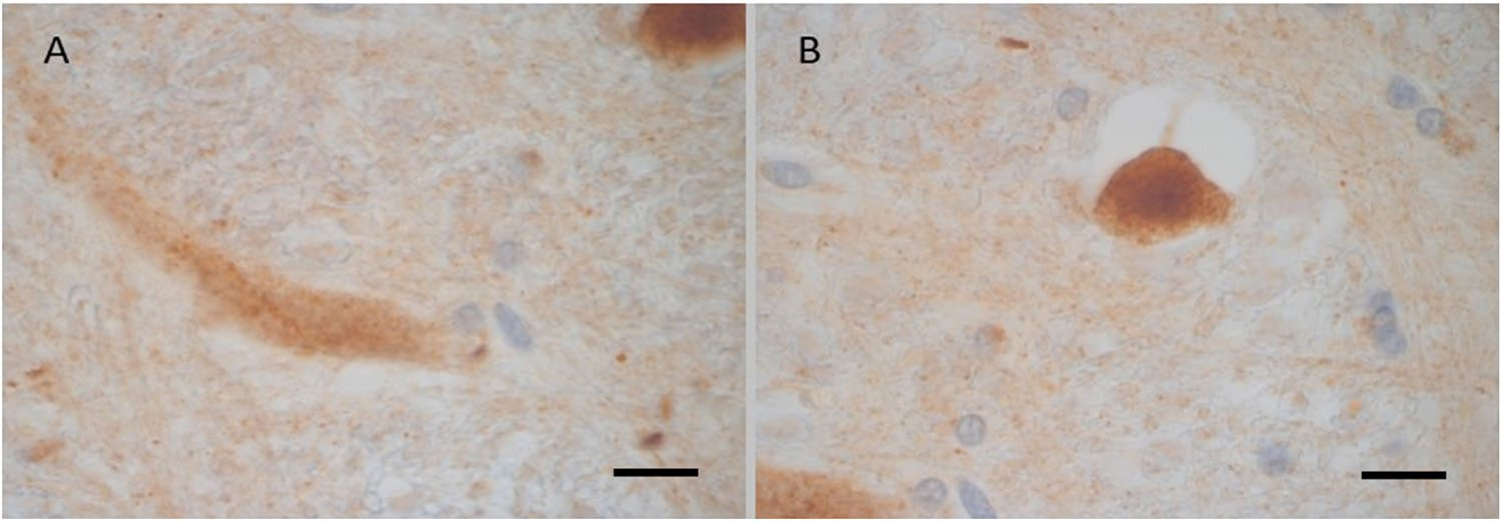
Case 3 group 3. 16-year-old male. Unrestrained passenger ejected from his seat during an RTC. Taken to hospital with a skull fracture, SAH, and SDH. Died in the hospital 3 days after incident

The βAPP is frequently and more intensely deposited (+ + , +  + +) in all regions of those with head injury in more than one region but less so in the brain from the patient who survived 31 days ( Table 2). The βAPP was seen as rounded or fusiform globules, or beaded and thickened filamentous structures, some with faint and granular staining (Fig. 4). The βAPP was only occasionally deposited in ill-defined focus of a granular pattern in one region in one of patients who suffered from ischaemia but without no clear linear ( geographical) pattern and is negative in the other one.

### Group 4 (control)

**Fig. 5:**
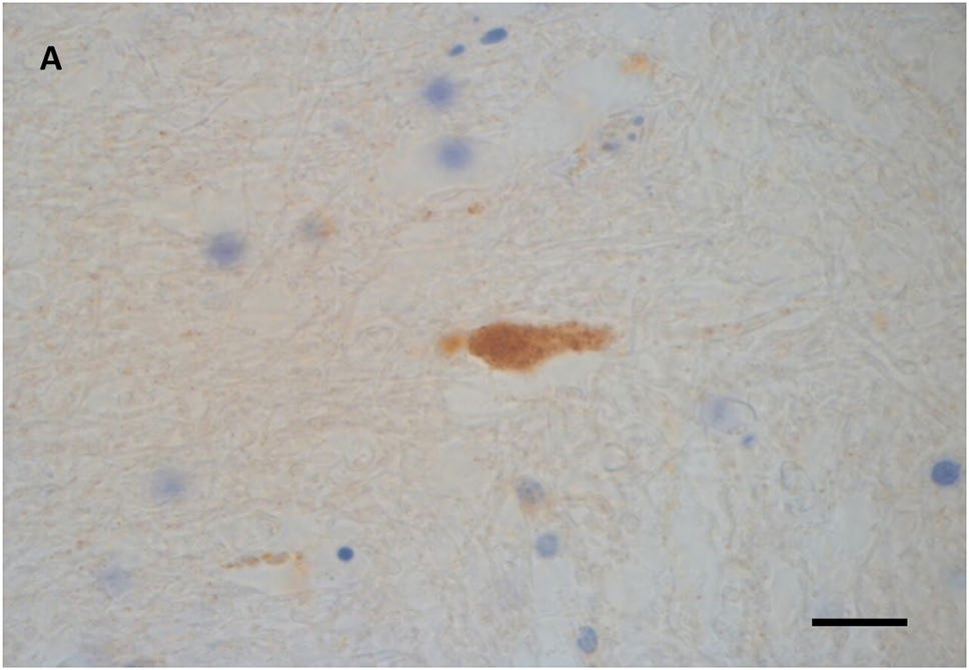
Case of 72-year-old female patient who was diagnosed with metastatic carcinoma of unknown origin to the liver and lung. She died suddenly and unexpectedly after being observed well an hour earlier. There is a single spot of βAPP accumulation as small globules with faint and granular staining in the cerebral white matter consistent with a non-specific axonal injury. The βAPP was negative in other examined regions in the brain

The βAPP was negative in all stained regions in brains from the four patients who died from sudden unexpected death in epilepsy and two patients who died suddenly and unexpectedly with underlying cancer. In one region of white matter of frontal lobe from brain of 72-year-old patient (control case number 5 Table 2 and Fig. 5), there was one small accumulated of deposit βAPP as small globule with faint and granular staining pattern consistent with non-specific axonal disruption. This staining pattern is different from the pattern and intensity and frequency seen in βAPP deposits seen in group 1, 2, and 3.

There were no other deposits, particularly in the regions of corpus callosum, internal capsule and brainstem which are more susceptible to axonal damage in cases of traumatic brain injury.

## Discussion

The previous work by Hortobagyi et al*.,* 2007 had provided evidence that using βAPP immunohistochemistry can detect axonal damage within 35 min after severe head injury [20]. In the current study however, we are able to demonstrate that in fact axonal injury can be detected earlier than this time with βAPP staining in patients who died rapidly at the scene of an RTC (i.e., in a few to several minutes but less than 30 minutes after the accident). This is an interesting and important finding of medicolegal importance, which is useful in the diagnosis of head injury with very short survival time.

One possible explanation as to why we are able to detect βAPP accumulation earlier than the previous studies is the method of identification of the time of death and the post-traumatic survival time. This is known to be a difficult issue to ascertain in forensic practice. In the Hortobagyi et al*.* study, they depended on ascertaining the time of death by when it was officially recorded by qualified paramedics after arriving at the scene of the incident, therefore death could have occurred earlier than they stated and in less than the 35 min. In contrast, in the current study, we were able to estimate the time of death from the prehospital emergency services information provided to us. Where time had elapsed between the initial call and the first medical responder arriving on scene finding the patient in cardiorespiratory arrest, the time of death was considered as have occurred shortly after the trauma in matter of few to several minutes. This is based on the presence of injuries incompatible with a period of survival of more than few minutes at most or even instantaneous, such as decapitation, complete spinal transection and those with minimal cavity bleeding, or the severity of blood loss (e.g., complete transection of aorta and avulsion of heart) or presence of airway/ventilation compromising injuries such as haemothorax, haemopericardium, haemopertioneum, tension pneumothorax or tension pneumopericardium (rapid cardiorespiratory arrest within minutes of trauma).

Another important point of difference is that Hortobagyi et al*.* studied only seven cases of head injuries resulting from assaults [20], while our study involved a larger number of fatal head injuries resulting from RTC. Therefore, another possible explanation as to why we were able to detect βAPP in shorter survival times is that our cases represented a different cohort of more severe head injury resulting from RTC. It is expected that the magnitude of the force in RTCs and the severity of acceleration and deceleration of the brain tissue would be more severe compared with those resulting from assault [8, 25–28]. This issue can be further clarified with an understanding of the pathophysiology of the axonal injury and the physiological function of βAPP and in appreciating the heterogeneous types of TBI and its different effects on varying brain regions.

There are also technical differences between the previous of Hortobagyi et al. and the current work regarding the detection of βAPP. We used a different antibody with a well-calibrated dilution and standardized staining using an auto-stainer, compared with previously used manual methods. Although it is possible that these technical differences could have improved the ability to detect βAPP, we believe they have minimal effect on overall results compared with the other factors such as the nature of the different cohort of head injury cases mentioned above.

It has become clear that traumatic axonal damage is common in all degrees of head injuries but the severity varies in different cases depending on the load of force and degree of acceleration and deceleration of the brain—which are directly related to the mechanically altered axons [29].

At one end of the spectrum, in mild-to-moderate head injury there may be mild-or-temporary transmembrane ionic disturbance of axons which could be reversible. This form of axonal injury is characterized by increased axolemmal permeability, mitochondrial swelling and cytoskeletal compaction disturbing the microtubules and neurofilament structure [30, 31]. The mild-to-moderate axonal injury is not necessarily associated with axonal transection but may cause reverse axonal transport which has been considered as a possible mechanism for accumulation of the transported axonal products, including βAPP [32].

At the other end of the spectrum is severe head injury, causing immediate primary axotomy or marked disruption of the axonal cytoskeleton ending in secondary axotomy. In the injured axon, the increase in the permeability of axolemma is more significant and causes more marked alterations in transmembrane ion concertation accompanied by fluid entry to axons leading to axonal swelling [33, 34]. With more severe injury, the extent of intercellular Ca ion accumulation leads to proteolysis, rapid compaction of neurofilaments and later collapse of the cytoskeleton [35, 36]. If the cytoskeleton cannot remodel itself, then delayed secondary axotomy will eventually occur leading to irreversible damage. This is an active energy-dependent process and may take a few minutes to several hours if the patient survives, depending on the magnitude of the loaded forces and the type of axons and location. This form of secondary delayed axonopathy that occurs subsequent to traumas had been characterized by progression of the formation of axonal balls and varicosities thought to be within one hour after injuries with disconnection by about 6 to 12 h and which can be easily detected by immunoreactivity of βAPP [37]. In cases of very severe head injury such as those resulting from RTC, the shearing of the axonal fibers leads to complete disconnection in a process of primary axotomy. However, despite the apparent correlation between the magnitude of the head injury and the traumatic axonal injury, primary axotomy is thought to be a minor contributor compared with the secondary mechanism in overall axonopathy seen in TBI [34, 35]. Nevertheless, it is still possible that very severe head injury, such as those caused by RTC, may cause a significant axonal injury and rapid accumulation of the transported products in the injured axons, including the ΒAPP, leading to the formation of the small individually well-defined swellings as seen in our cases. These most likely represent morphologically normal but functionally incapacitated axons with disruption of normal axoplasmic flow [38].

In few cases of group 1, the death is expected to be very rapid such as case number 10 in which the death had resulted from severe polytrauma including severe head injuries with multiple skull fractures resulted from a fatal airplane crash. Despite the expected very rapid death, there are infrequent βAPP immunoreactive foci in the cerebral white matter, corpus callosum and cerebellar white matter which suggest that βAPP can be accumulated in tiny amount even with low level of blood circulation in such conditions considering the fact that βAPP is active energy-dependent process.

Further support for our suggestion comes from in vitro and animal studies: In vitro studies using neuronal preparations reveal that immediately subsequent to stretch injuries the axonal structure is distorted, and some axons become undulated and twisted due to cytoskeletal damage. The internodal regions of the axons appear to be particularly vulnerable, whether due to specific mechanical features of this region or lack of association with supportive astrocytes. The changes in the cytoskeleton and neurofilament structure had been detected within 15 min after injury with subsequent disruption of the axonal transport and accumulation of the transport product [28, 39, 40].

Although animal models may not exactly replicate the reaction to traumatic axonal injury, they are useful to shed light on the mechanism and timing of damage. These models demonstrate ultra-structural changes detectable within few minutes of injury. These include mitochondrial swellings followed by misalignment of microtubules which inevitably leads to distortion of ante-grade and retro grade axonal flow interfering with normal transport of products down the axons [28, 41].

We consider the possibility that some of the axonal disruption in group 1 patients may be present prior the RTC such as that caused by alcohol, drug overdose or aging but we believe this possibility is less likely (although cannot be completely excluded) because the pattern and intensity of the βAPP would be seen as more intense, widespread ill-defined granular deposits—different from the described pattern in group 1. This is well demonstrated in the control brain of age 72 years (case number 5 of group 4 in Table 2). There is one small globule in the white matter with faint and granular staining ( Fig. 5) which is different from the more densely stained small globule seen in group 1 cases.

We propose that if the head injury is severe, like those in severe and fatal impact injury, one would expect a severe and rapid axonal damage, probably more frequent primary axotomy or a very rapid secondary axotomy occurring in minutes. The neurofilament backbone of the axons quickly collapses, leading to disintegration at the site of the damage. Following this, the axolemma beginnings to fold inward and separate from the underlying myelin sheath. The transported proteins continue down the stream of the axoplasm until they reach the site of injury where they immediately accumulate because they are unable to progress. This is the point when βAPP is expected to be visible as small well-defined globules or thin filaments.

Another possible explanation as to why βAPP can identify the site of axonal damage so early after trauma may be based on the function of the βAPP protein as a rapid post-traumatic inflammatory and neuroprotective response. TBI is an intrinsically heterogeneous process that does not occur in vacuum. It in fact causes multiple cascades of events involving upregulation of oxidative, excitoxic and inflammatory pathways that affect the evolution of axonal pathology [42, 43]. βAPP is a ubiquitous membrane glycoprotein produced in the cell body and it is transported in vesicles by fast anterograde axoplasmic transport as it has synaptic function. It plays a physiological role in cell adhesion, functions as a contact receptor, synaptic modification and endogenous neuroprotection in response to injury such as against ischaemia and excitotoxic injury [21, 44]. Its activation and overexpression may be part of an immediate inflammatory neuro-protection response to injury together with many other factors [21, 45]. One of these factors is clusterin which is specifically explored by Troakes et al., in cases of head injury [46]. Clusterin is also known as apolipoprotein J (ApoJ), a highly conserved ubiquitous glycoprotein in the brain. It is a stress-induced chaperone which is normally secreted; however, in conditions of cellular stress it can be transported to the cytoplasm where it binds to Bax protein and inhibits neuronal apoptosis [47]. Additionally, clusterin is a well-known complement inhibitor, binding to C5b-9 component and inhibiting the formation and membrane binding of MAC (membrane attack complex) [48, 49].

Troakes et al*.* have shown that clusterin is over-expressed very soon after the onset of TBI, suggesting that it plays a role in the response to blunt trauma and that it may assist in remodeling and modulation of the inflammatory response following this form of brain injury. They also demonstrated that staining of clusterin was closely associated with βAPP accumulation when examined by double immunofluorescence in white matter, which is consistent with overexpression at the site of axonal injury and dysfunction. Clusterin was observed deposited in the white matter of brains from patients who died from head injury in less than 30 min after the initial injury, although as previously mentioned it is quite possible that death could have occurred even earlier and that clusterin is deposited extremely rapidly after TBI [46].

If the severe head injury in RTC can cause marked and widespread mechanical stress and expected widespread axonal injury, then why does the immunohistochemical staining with βAPP only reveal infrequent and scattered sites of axonopathy? One explanation is that the myelination varies between neighboring axons and that finely myelinated small axons appear more vulnerable to injury [28]. It is therefore likely that the visible microscopic findings of BAPP accumulation underestimate the extent of injury in the neighboring fibers with varying degree of injury as the proportion of histologically abnormal axons seen in trauma models and patients is inconsistent with the magnitude of the TBI. Another possibility is that the mechanical load of the transmitted forces is variable throughout the brain regions and may have variable effect on different types of axons (diameter, length and myelination) leading to different spectrum of changes in different brain regions.

As mentioned earlier, the very early accumulation of BAPP in axonal injury in patients who died at the scene of an RTC indicates interruption of the fast-axonal transport and not necessarily axotomy. Some βAPP may accumulate due to temporary and reversible axonal dysfunction [3]. Therefore, from a practical point of view, it may not be possible to know the significance of this accumulation in relation to the degree of head injury. However, the involvement of infra tentorial parts of the brain including the cerebellum, cerebellar peduncles and brain stem structure in additions to the supra tentorial parts would raise possibility of a severe degree of head injury [3, 20]. Nevertheless, care should be taking not to over interpret the small amounts of βAPP accumulation in rapid death after head trauma without other evidence of severe head injury. Another note of caution in the same issue is to consider the possibility of an ischaemic disruption of axons accompanying the head injury [19, 50]. The ischaemia in the context of RTCs is frequent resulting from reduction of the cerebral blood flow due to other systemic injuries and blood loss. In our cohort of RTC, the ischaemia could have a contribution to the axonal injuries and accumulation of βAPP but this is extremely difficult to ascertain or disprove in this work which actually aimed to investigate the timing of the axonal injury due to head injury or its consequent ischaemia.

There is no data as to when βAPP accumulation may be expected to appear in ischaemia alone. One would expect it to be of similar timing to those of trauma but this is not necessarily true. Ischaemia is not expected to cause primary axotomy or rapid disruption to the axonal cytoskeleton as seen with trauma, therefore it is less likely to cause such rapid accumulation of βAPP [19].

The progression and evolution of the axonal injury reflected by the shape and the frequency and intensity of βAPP accumulation after hours and days survival time (group 2 and 3) is well demonstrated in this and confirms the findings in previous studies. We found the amount and frequency of staining of βAPP in head injuries increases with the increasing time of survival of the victim and increased intensity reflecting larger amounts of accumulated protein in group 2 and group 3. When patients survived a few days, the intensity became less and the βAPP staining became faintly granular, almost disappearing after one-month survival (case number 45).

## Summary

Axonal injury can be detected in the brain of those who died rapidly following fatal road traffic collision. We propose that the early accumulation of βAPP is probably due to the high magnitude of loaded forces in head injury resulting from RTC in this cohort which could possibly cause higher proportions of primary axotomies and/or that the stretching axonal injuries are more severe, causing quicker alteration in the axonal cytoskeleton. Our result is expected to have implications on the timing of head injuries in medicolegal practice but caution is recommended so as not to over-interpret the results which require close correlation with autopsy findings, circumstance of the incidence, and other investigations.

## Appendix

### Tissue preparation and immunohistochemistry

Formalin-fixed, paraffin-embedded tissue (FFPE) blocks were used for this investigation. Brain autopsy samples were fixed in 10% formalin for a minimum of 48 h to allow adequate preservation of cells and antigens to obtain reliable immunohistochemical (IHC) staining. Tissue processing and immunohistochemical stains were then carried out following standard methods following the main four steps, fixation, dehydration, clearing and wax impregnation.

Paraffin wax sections were cut at 7 µm and mounted onto positive charge coated microscope slides for routine Haematoxylin & Eosin (H&E) staining and IHC staining with βAPP.

### IHC staining

All sections produced were stained with βAPP by means of automated IHC staining, using the Leica bond platforms. These machines follow the labelled polymer method with DAB as the chromogen causing positive staining to present itself as brown. The staining protocol has been established by the Department of Clinical Neuropathology at Kings College London Hospital and has been tested and validated for diagnostic purposes, determining the most appropriate antibody dilution as well as antigen retrieval method.

Sections were dewaxed on the Leica bondmax machines followed by the heat-induced epitope retrieval (HIER) for 20 min in epitope retrieval 1 solution (ER1), a pH 6.0 citrate buffer. The primary antibody, βAPP, was then applied for 15 min (Millipore MAB348 diluted to 1/5000). A polymer refine detection kit was used to complete labelling. These kits contain all the necessary components to complete the staining technique including peroxidase block, post primary, polymer reagent and the DAB chromogen.
